# Graphene functionalised by laser-ablated V_2_O_5_ for a highly sensitive NH_3_ sensor

**DOI:** 10.3762/bjnano.8.61

**Published:** 2017-03-07

**Authors:** Margus Kodu, Artjom Berholts, Tauno Kahro, Mati Kook, Peeter Ritslaid, Helina Seemen, Tea Avarmaa, Harry Alles, Raivo Jaaniso

**Affiliations:** 1 Institute of Physics, University of Tartu, W. Ostwald Street 1, EE50411 Tartu, Estonia

**Keywords:** ammonia, electric conductivity, gas sensor, graphene, pulsed laser deposition, UV light activation, vanadium(V) oxide

## Abstract

Graphene has been recognized as a promising gas sensing material. The response of graphene-based sensors can be radically improved by introducing defects in graphene using, for example, metal or metal oxide nanoparticles. We have functionalised CVD grown, single-layer graphene by applying pulsed laser deposition (PLD) of V_2_O_5_ which resulted in a thin V_2_O_5_ layer on graphene with average thickness of ≈0.6 nm. From Raman spectroscopy, it was concluded that the PLD process also induced defects in graphene. Compared to unmodified graphene, the obtained chemiresistive sensor showed considerable improvement of sensing ammonia at room temperature. In addition, the response time, sensitivity and reversibility were essentially enhanced due to graphene functionalisation by laser deposited V_2_O_5_. This can be explained by an increased surface density of gas adsorption sites introduced by high energy atoms in laser ablation plasma and formation of nanophase boundaries between deposited V_2_O_5_ and graphene.

## Introduction

Graphene, being a thin (semi)conducting material, is a promising gas sensing system. Highly sensitive response, down to single molecule resolution, has been demonstrated with graphene-based devices under laboratory conditions [1–3]. However, in order to develop gas sensing applications working under real conditions, much effort has been dedicated to modification of graphene for improving its gas sensing characteristics. In particular, increasing the selectivity of graphene-based gas sensors is crucial for their future implementation. Recently, the improvement of gas sensing characteristics has been demonstrated with resistive type gas sensors based on single-layer graphene modified by a deposited layer of precious metal [4] or metal oxide nanoparticles [5]. Also, introduction of suitable defects was shown to have a positive effect on gas adsorption and sensor properties of graphene [6].

Transition metal oxides constitute an important class of catalysts and photosensitizers. Apart from the very first and last 3d elements, scandium and zinc, the rest of the metals possess several oxidation states. The presence of several stable oxidation states serves as a basis of catalytic activity in redox reactions and is most noticeable for vanadium, chromium, and manganese. In particular, vanadium has the highest oxidation state in vanadium pentoxide (V_2_O_5_) – a good oxygen transfer catalyst that is (thermally) stable in air and vacuum [7–10]. Therefore, we considered vanadium oxide as a promising material for functionalising a graphene sensor in order to increase its selectivity towards reducing pollutant gases, such as ammonia. Vanadium oxide based films and nanostructured layers have been previously synthesised for gas sensing applications by various methods [11], including pulsed laser deposition (PLD) [12].

PLD is a highly versatile method for relatively well-controlled preparation of thin films, and many advanced composite materials have been produced for diverse applications [13]. The possibility to evaporate practically any solid material, tune the kinetic energy of particles between 0.1 to 1000 eV, as well as the ability to control the amount of deposited material from about 1/100th of a monolayer per laser pulse are the advantages worth mentioning. The method of PLD has recently been applied to improve the nitrogen dioxide (NO_2_) sensing properties of chemical vapour deposition (CVD) grown, single-layer graphene in our previous work, using ZrO_2_ and Ag for functionalisation [14].

In the present work, we demonstrate functionalisation of single-layer CVD graphene with a few layers of laser deposited V_2_O_5_. The amount and chemical state of vanadium oxide on graphene was characterized by X-ray photoelectron spectroscopy and X-ray fluorescence. The impact of the PLD process on graphene defect structures was investigated using Raman spectroscopy. Based on the electrical conductivity modulation, the room temperature gas sensing properties of the manufactured sensor structure towards ammonia (NH_3_) and (for comparison) nitrogen dioxide (NO_2_) gases were investigated.

## Results

**Figure 1:**
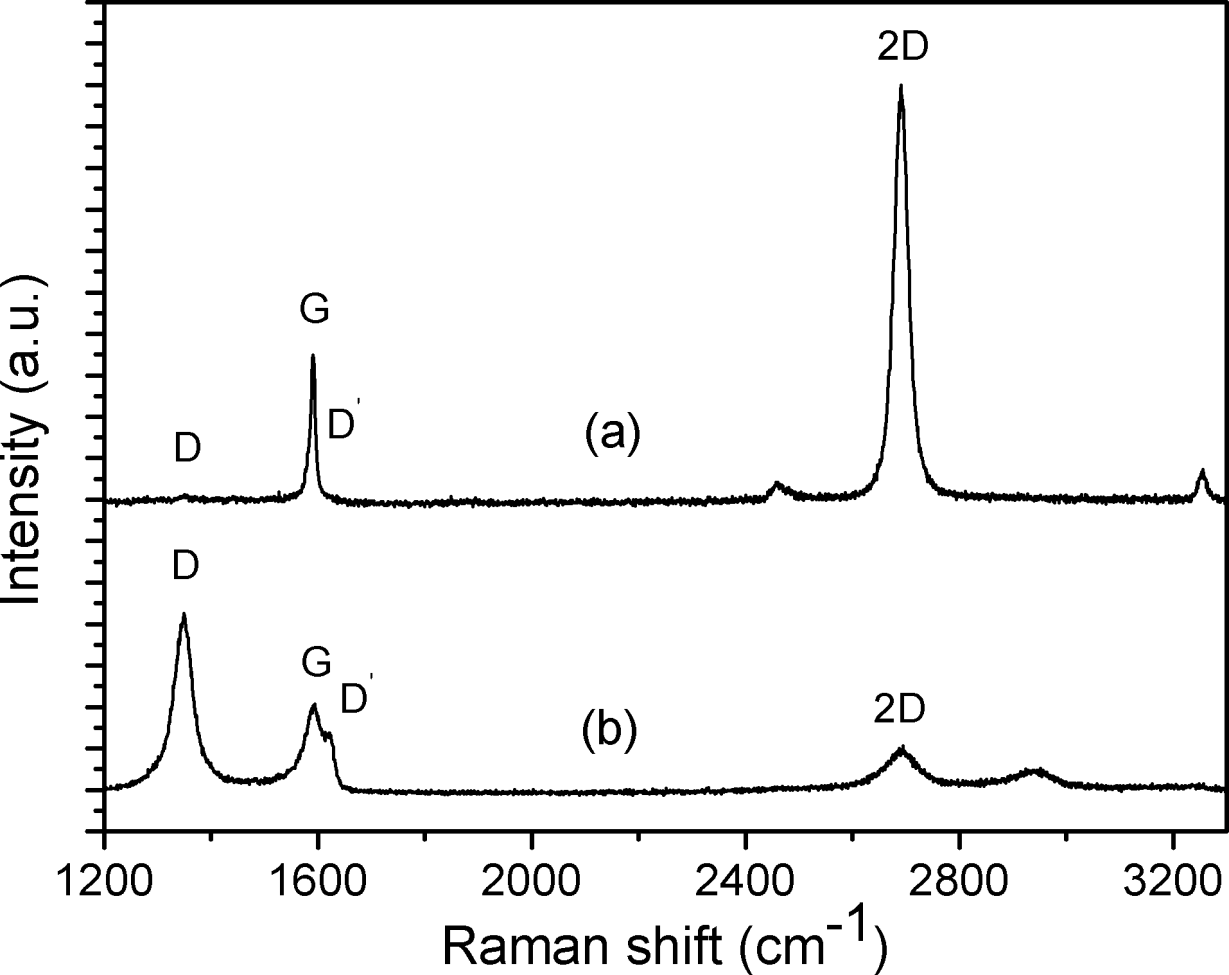
Typical Raman spectra of the graphene sensor device recorded between electrodes (a) before and (b) after laser deposition of V_2_O_5_. Defect-related peaks, D and D’, are absent in (a).

Figure 1a shows a typical Raman spectrum from graphene placed between the electrodes of the gas sensor structure (see Experimental section). The G and 2D bands peak at ≈1590 cm^−1^ and ≈2690 cm^−1^, and have full-widths at half-maximum of 11 cm^−1^ and 29 cm^−1^, respectively. These characteristics, together with the ratio of G to 2D band peak intensities of approximately 1:3, correspond to single-layer graphene [15]. The absence of a defect-related D band at ≈1350 cm^−1^ indicates extremely low defect density [15–16]. After the PLD of V_2_O_5_ onto graphene (for the PLD details, see the Experimental section below), the defect-related D and D’ bands emerge in the Raman spectrum, and at the same time, the G and 2D bands decrease in intensity (Figure 1b).

**Figure 2:**
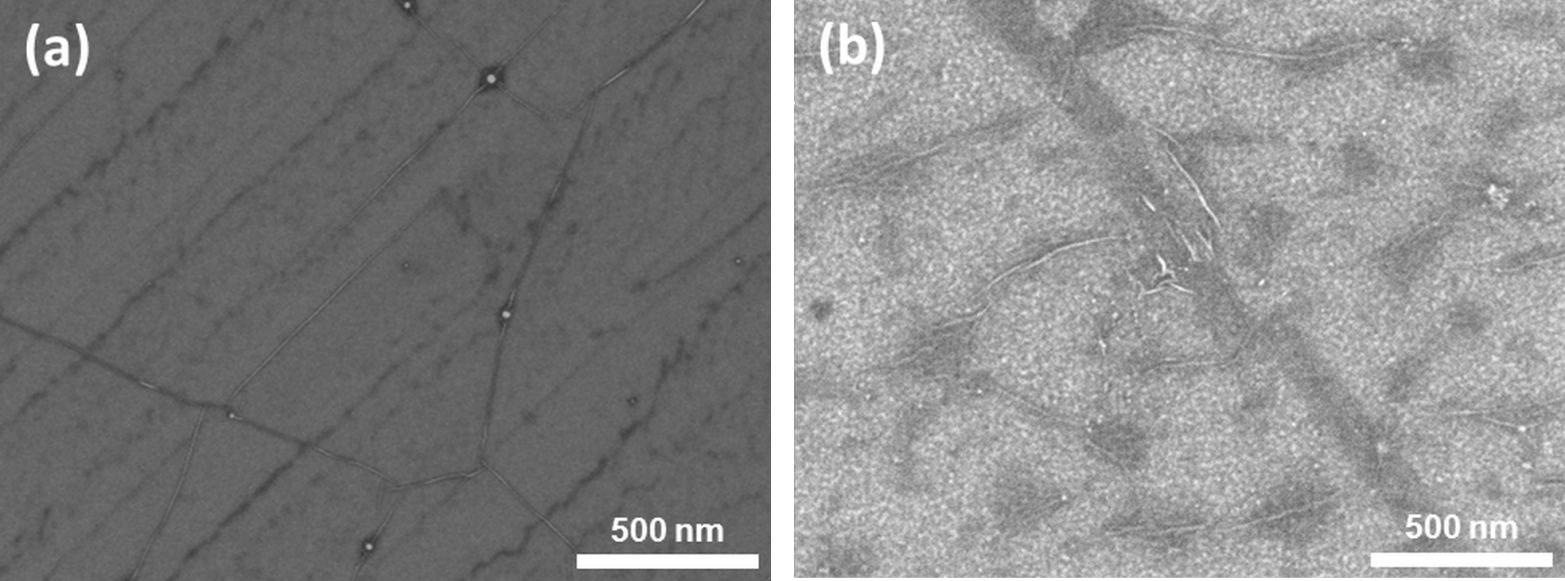
SEM images of a pristine single-layer graphene surface (a) and graphene functionalised by V_2_O_5_ (b).

Figure 2 shows scanning electron microscope (SEM) images of the graphene surface before and after PLD of V_2_O_5_. The darker contrasting regions and the lines in the image originate from the Cu growth surface of the CVD process or wrinkles left in the graphene sheet during the transfer process from the copper foil to Si/SiO_2_ substrate. These features are characteristic for CVD graphene and can also be seen in the SEM image of pristine graphene shown in Figure 2a. The islands of about ≈20 nm in diameter from the laser-deposited nanostructured material can be distinguished in the image. It is well known that gas phase species created by laser ablation of solids have a wide distribution of kinetic energy [17]. A considerable fraction of particles can have sufficient energy (≈100 eV) for creation of point defects in the graphene sheet [18]. As a consequence, the Raman lines assignable to point defects or imperfect graphene edges appear. The extent of disorder induced by the PLD process is characterized by the ratio of Raman line intensities *I*_D_/*I*_G_. Considering the ratio *I*_D_/*I*_G_ = 2.13 obtained from Figure 1, and applying the formulae given by Piment et al. [19] and Concado et al. [20], estimates are obtained for the average graphene crystallite size and the distance between point defects of ≈7.9 nm and ≈7.7 nm, respectively.

The amount of vanadium that was deposited on the sensor substrate was evaluated using X-ray fluorescence (XRF) analysis by measuring the amount of vanadium in the film deposited onto a fused quartz substrate under the identical PLD procedure. According to XRF, the mass thickness of vanadium on the substrate was 0.11 µg/cm^2^. Presuming that all of the vanadium on the substrate belongs to the composition of V_2_O_5_, and considering the density of crystalline V_2_O_5_ of 3.38 g/cm^3^ [21], an estimate for the average thickness of a continuous V_2_O_5_ layer on graphene is 0.58 nm. In other words, the approximate number of V_2_O_5_ (001) lattice plane layers is about 2.5.

**Figure 3:**
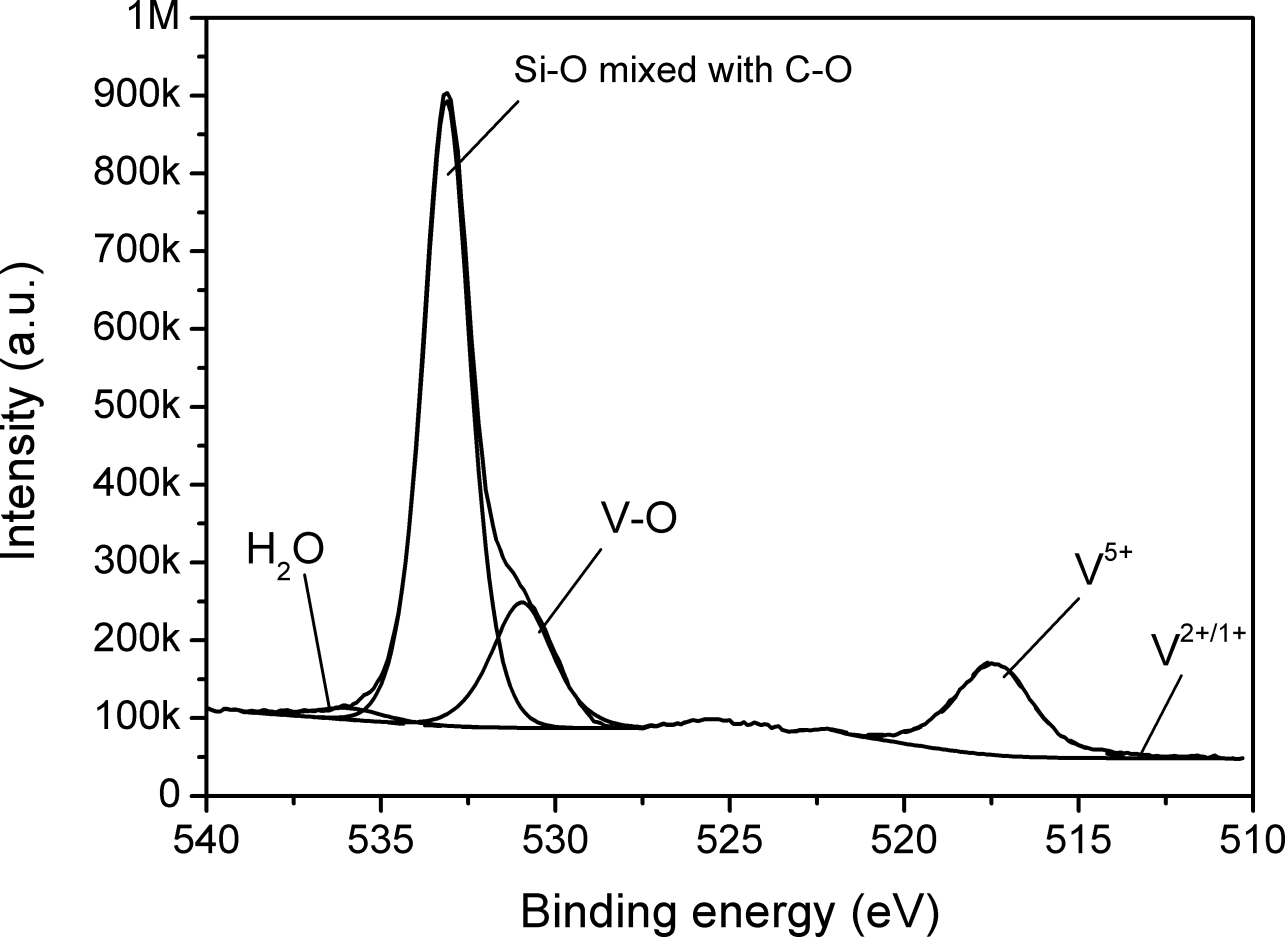
XPS spectra of graphene following PLD treatment with V_2_O_5_ in the O 1s and V 2p region. A polychromatic Mg Kα source was used for excitation and the satellite peaks were removed.

The oxidation state of vanadium was determined by means of X-ray photoelectron spectroscopy (XPS). Figure 3 depicts the O 1s and V 2p regions in the XPS spectrum measured from the surface of the sample after the PLD process. Because the source was not monochromatic, the V 2p_1/2_ region is not usable for the analysis, owing to the overlap with the Mg Kβ satellite with O 1s peak. The main component of V 2p_3/2_ at a binding energy of 517.4 eV was assigned to V^5+^, whereas a faint component at 513.3 eV belongs to V^2+/1+^. The O 1s peak component at 530.9 eV was identified as oxygen bound to vanadium, in agreement with Biesinger’s XPS measurements on vanadium oxide [22]. The main peak component of O 1s at 533.1 eV was attributed to the oxygen in SiO_2_ in the silica substrate that can possibly overlap with the C–O component of (partly oxidized) graphene at the same binding energy (533.1 eV) [23]. Thus, according to XPS and XRF analysis, the material deposited on graphene is predominately V_2_O_5_, with an average thickness of about 0.6 nm. Among other vanadium oxides, V_2_O_5_ is a relatively stable compound that can be easily deposited by the PLD method [12].

**Figure 4:**
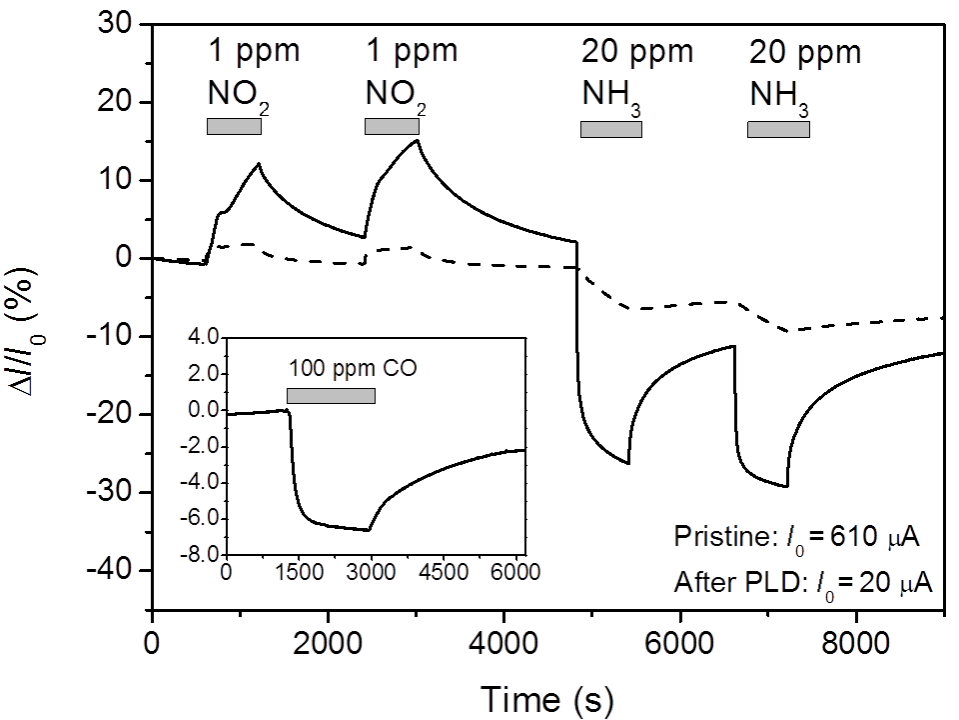
Electrical conduction response of pristine graphene (dashed line) and graphene functionalised with a V_2_O_5_ layer (solid line) with respect to NO_2_ and NH_3_ gases. The measurements were performed at room temperature under UV illumination. The horizontal bars indicate the time intervals of gas exposure. The inset depicts a response to 100 ppm CO gas.

Figure 4 depicts the time response of a PLD-functionalised graphene sensor to polluting gases NO_2_ and NH_3_. All the gas measurements in this work were recorded under continuous excitation with ultraviolet (UV) light (λ = 365 nm) at room temperature (RT). Illumination by UV light can enhance the sensing performance of graphene-based gas sensors. An increased gas response and fast recovery under UV irradiation has been demonstrated for pristine and functionalised graphene-based sensors, possibly as a result of cleaning the surface of interfering or passivating gases [24–26]. The effect has been explained by photo-induced desorption of oxygen and water molecules, thus activating additional adsorption sites on graphene for the target gas.

It should be noted that after the PLD of V_2_O_5_, the conductivity of the sensor decreases by a factor of 30. According to the Raman spectra (Figure 1), the PLD process is fairly destructive and induces a high concentration of defects to the graphene 2D crystal lattice. These defects decrease the charge carrier mobility in graphene which is reflected in reduced electrical conductivity.

As compared to the pristine sensor, the response to both gases is clearly improved after the functionalisation by PLD. The response to 1 ppm NO_2_ and 20 ppm NH_3_ increases from 2 to 12%, and from 6.5 to 26%, respectively. The sensor current shows a clear-cut and reversible response to the test gases NO_2_ and NH_3_. As shown in Figure 4, the sign of the conductivity of the graphene sensor changes after introducing another gas (NO_2_ or NH_3_) into the test chamber. Graphene is typically a p-type conductor under ambient conditions due to chemical doping by adsorbed oxygen and water molecules [27–28]. Bearing in mind that NH_3_ acts as a hole acceptor and NO_2_ as a hole donor [1], the conductivity is expected to increase or decrease, respectively, which is indeed observed. The response to another common reducing gas, the air pollutant carbon monoxide (CO) was also tested. The inset in Figure 4 demonstrates the response of a V_2_O_5_ functionalised sensor to 100 ppm CO. Although the sensor is sensitive to CO and a ≈7% decrease in conductivity is observed, the sensitivity to CO is much lower than to NH_3_.

**Figure 5:**
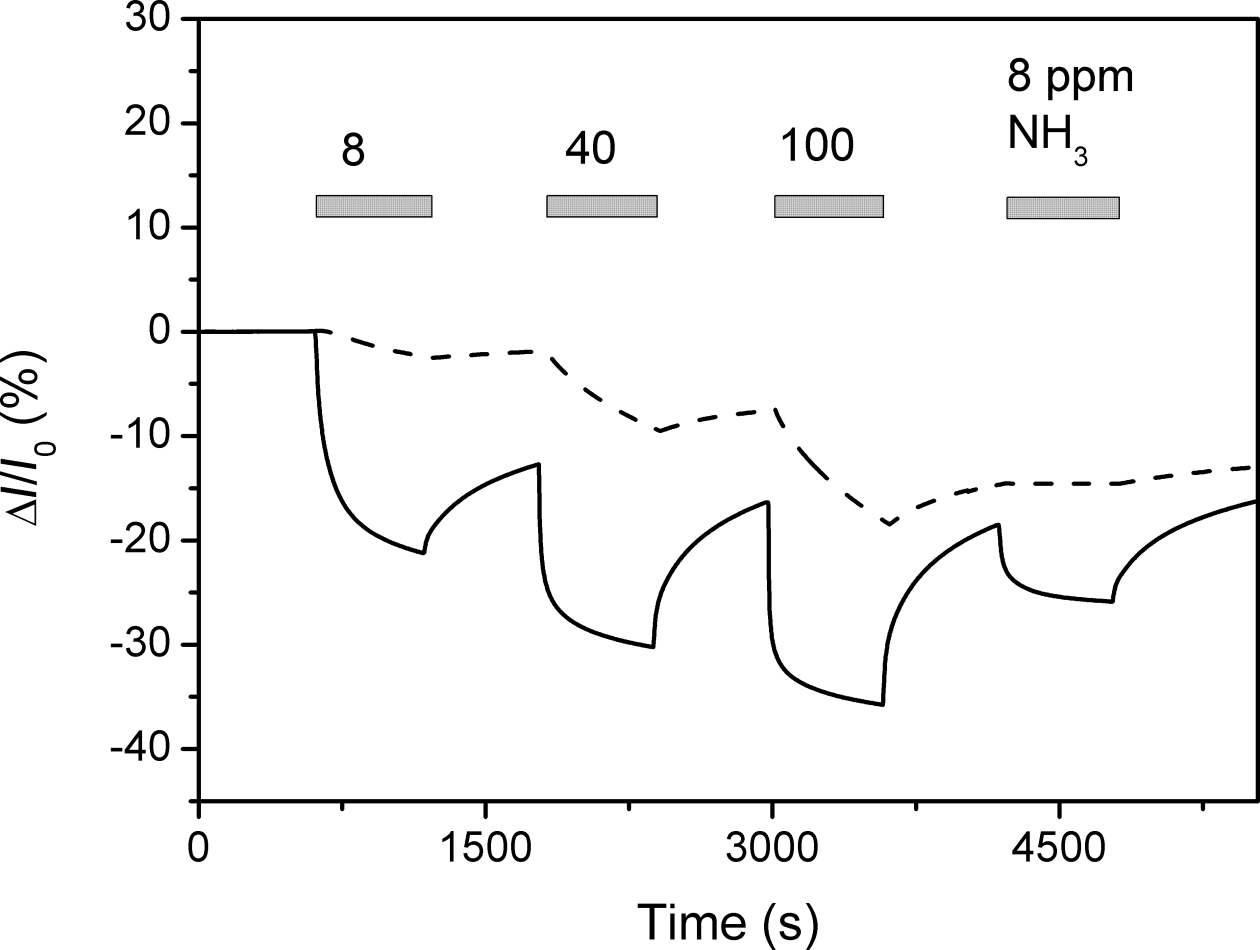
Response of the graphene sensor to different concentrations of NH_3_ gas before (dashed line) and after (solid line) PLD functionalisation, measured under UV illumination at the room temperature. The grey bars indicate the duration of gas exposure for the given concentration.

Figure 5 illustrates the effect of laser-deposited V_2_O_5_ under NH_3_ gas at different gas concentrations. The functionalisation with V_2_O_5_ clearly improves the performance of the sensor. The response and recovery times of pristine and functionalised sensors for 8 ppm NH_3_ gas were determined by fitting the time curves with the suitable functions. Either single or double exponential functions were used in the following form:

[1]
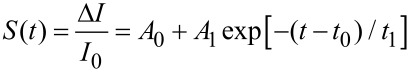


[2]
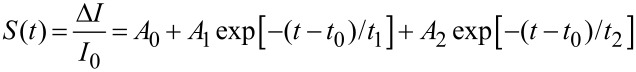


In Equation 1 and Equation 2, *S*(*t*) is the relative change of conductance and *t*_0_ is the initial time when the respective stepwise change in gas composition was introduced. The characteristic times *t*_1_ and *t*_2_ are related to the rate constants of adsorption and desorption of gas molecules onto pristine or V_2_O_5_-functionalised graphene. In principle, a single exponential type of response (Equation 1) corresponds to the case where there is only one type of adsorption site available for adsorbing molecules at the surface of the graphene sensor. Similarly, the double exponential type of response (Equation 2) may describe the situation where two sites with different adsorption and desorption rate constants are available. The physical aspect of modelling the graphene sensor response is discussed more exhaustively by Jaaniso et al. [29].

In the case of pristine graphene, the response and recovery could be fitted with a single exponential function (Equation 1), which yielded characteristic times 578 s and 738 s for the response and recovery, respectively. In the case of V_2_O_5_-functionalised graphene, the response could be fitted with the double exponential function (Equation 2), with characteristic times 25 s and 175 s for the response, and 42 s and 442 s for the recovery. Although the response and recovery kinetics of the functionalised sensor is double-exponential, it is safe to conclude that both the sensor response and recovery times become significantly faster as a result of the laser deposition of V_2_O_5_ – the response increased by ≈3 times and the recovery by ≈2 times for the functionalised sensor. The amplitude of the response during the 600 s gas exposure increases by 2 times for a gas concentration of 100 ppm, and by 8 times for 8 ppm NH_3_. This suggests that the sensitivity is more significantly improved at lower concentrations.

## Discussion

Perfect graphene is relatively inert because of the lack of dangling bonds and charged atoms on the surface. Consequently, the adsorption energy is due to van der Waals forces, and may be less or comparable to *k*_B_*T* (where *k*_B_ is the Boltzmann constant and *T* the absolute temperature) for gases at room temperature. The introduction of defects and dopant atoms into graphene can drastically increase both the adsorption of pollutant molecules and the influence of gas adsorption on the electronic properties of graphene [6,30]. For instance, adsorption energy (*E*_a_) of an NH_3_ molecule on regular graphene is relatively small (*E*_a_ ≤ 0.11 eV [6,30]), but it is much higher for defect (up to 1.5 eV [6,31]) or impurity-doped graphene (up to 1.4 eV [6,30,32]). The introduction of defects and doping atoms into graphene sheets is necessary to enhance the interaction between the target gas molecules and graphene. However, this also results in increased interaction of H_2_O and O_2_ molecules with graphene [30]. This would result in the situation where active desorption sites are already occupied by strongly bonding O_2_ and H_2_O and the response to target gas would be slow and small. In some papers, UV light or annealing in vacuum is used to clean the surface of graphene from strongly adsorbed gas molecules, but during adsorption, the excitation is not used [1,26,31]. However, in practical applications there is a constant need to activate the sensor surface by cleaning it of H_2_O, O_2_ and other adsorbates, which would otherwise passivate it. The illumination by UV light as a tool for initiating or accelerating the desorption was introduced by Chen et al. [33] for carbon nanotube sensors. Thereafter, it was shown that the illumination is beneficial at the adsorption stage as well, acting to considerably enhance the magnitude of the response [34]. The benefits of UV excitation are also clearly observed for single-layer graphene functionalised by a thin layer of laser-deposited ZrO_2_ or Ag. First, under UV exposure, the sensor responses were at least 2 to 3 times faster. Second, without UV illumination, the recovery of the signal in pure air was almost absent in the case of ZrO_2_, and only partial recovery of the signal occurred in the case of Ag [14].

Generally, graphene-based sensors show much stronger response towards strongly oxidizing, paramagnetic (free radical) NO_2_ molecules than to any other gas, including NH_3_. The binding energy is large and intermolecular charge transfer can occur when NO_2_ adsorbs on either pristine, defective, or doped graphene [6,31]. Table 1 compares the NO_2_ and NH_3_ responses of our devices, and also of several other sensors reported in the literature, all based on a single-layer graphene. Only qualitative discussion is possible, because in most cases, the gas concentrations differ. It can be seen that most sensors are more responsive to NO_2_, as compared to NH_3_. However, concerning the relative responses, the results of Yavari et al. [35] are comparable to our V_2_O_5_-functionalised sensor. According to the measured Raman spectra, the CVD grown graphene used in these experiments contains a relatively large concentration of defects of unspecified nature [35]. The role of graphene defects in the case of NH_3_ sensing is well illustrated by Lee et al. [31], where the response to NH_3_ gas increased by 600% after creation of defects in the pristine defect-free graphene by reactive ion etching. At the same time, the response to NO_2_ gas increased only by 33% [31]. We would like to point out that the graphene sensors functionalised by PLD with Ag and ZrO_2_ in our previous work [14] showed a much larger response to 1 ppm NO_2_ than to 20 ppm NH_3_ (see Table 1, No. 2 and 3). Thus, decoration of graphene with V_2_O_5_ lends the sensor some degree of selectivity, and good sensitivity with respect to the reducing pollutant NH_3_.

**Table 1:** Comparison of the response of the V_2_O_5_-functionalised graphene (Gr) gas sensor to NO_2_ and NH_3_ with other sensors based on pristine and defect-induced graphene, or graphene functionalised with other materials.

No.	Material	Response to NO_2_	Response to NH_3_	Ref.

1	CVD Gr + V_2_O_5_	12% (1 ppm)	26% (20 ppm)	this work
2	CVD Gr + ZrO_2_	110% (1 ppm)	20% (20 ppm)	this work^a^
3	CVD Gr + Ag	20% (1 ppm)	11% (20 ppm)	this work^a^
4	exfoliated Gr	4% (1 ppm)	4% (1 ppm)	[1]
5	SiC/Gr + Au	55% (0.5 ppm)	20% (40 ppm)	[4]
6	defective CVD Gr	12% (2 ppm)	40% (40 ppm)	[35]
7	defective CVD Gr	53% (200 ppm)	25% (200 ppm)	[31]
8	B-doped CVD Gr	10% (0.02 ppm)	8% (20 ppm)	[36]

^a^Samples are described exhaustively in [14]. The responses to NH_3_ are measured in this work.

The introduction of dopants and clusters of atoms into graphene strongly increases the interaction with adsorbing molecules, due to an increased charge transfer and formation of chemical bonds between the dopant and adsorbate [6,32]. Moreover, changes in the electronic structure induced by adsorption of molecules are likely to modulate the conductivity of graphene [32].

According to the Raman analysis shown in Figure 1, the PLD process induces a large number of defects in graphene. We propose that during functionalisation of graphene by PLD, the defect creation in the graphene sheet by energetic plasma species is instantly followed by the V_2_O_5_ cluster growth at the defect site as the PLD process continues. The V_2_O_5_ clusters are probably chemically bound to the defect sites of the graphene sheet, yielding a strong and stable contact between the two phases. Chemical bonding is accompanied by charge transfer between V_2_O_5_ clusters and graphene. A similar situation was modelled previously by Lim and Wilcox [37] where large charge transfer and chemical bonding in platinum–graphene systems were observed when Pt nanoclusters were chemically bonded to point defects of graphene sheets. Furthermore, the adsorption of an O_2_ molecule onto a platinum nanocluster which was chemically bound to graphene resulted in a large influence on the charge density distribution of the system [37].

V_2_O_5_ is known for its catalytic properties and as a good NH_3_ adsorber [7–8]. The good NH_3_ gas sensing properties of V_2_O_5_ thin films and nanofibers have been demonstrated by Huotari et al. [12] and Modafferi et al. [38]. Two strongly bound adsorption species are typically observed as the result of the reaction of NH_3_ with two V_2_O_5_ adsorption sites, one with a surface OH group, forming positively charged NH_4_^+^, and the other with oxygen vacancy, forming species denoted as “coordinated NH_3_”. At the same time, the intensity of the V^5+^=O related band in the infrared reflectance spectra decreases, which is an indication of the reduction of V_2_O_5_ [7,9]. The corresponding ammonia oxidation reactions take place on the V_2_O_5_ catalytic surface. A variety of redox processes are possible, for example:

[3]
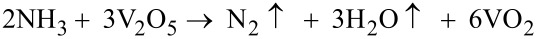


Ambient oxygen can render the process reversible, as desired for sensor reset, for example:

[4]
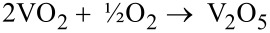


Obviously, strongly bound adsorbate species and the accompanying reduction of V_2_O_5_ can modify the charge distribution in the graphene–V_2_O_5_ system, thus modulating its conductivity.

According to Gao et al. [10], NO_2_ can adsorb to V_2_O_5_ to form nitrato groups (V^5+^–NO_3_), and this process is reversible:

[5]
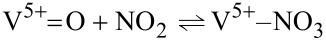


As there are no redox reactions involved in this adsorption process, the influence of NO_2_ adsorption on the conductivity of the V_2_O_5_–graphene system is lower, as expected. Additionally, other energetically favourable adsorption sites can exist at phase boundaries or at the point defects in the parts of graphene exposed to the gas, since the V_2_O_5_ material is on average only 2.5 layers thick and can cover the graphene surface unevenly.

## Conclusion

CVD graphene was functionalised by laser deposition of a sub-nanometre layer of catalytically active V_2_O_5_. The emergence of defect-related D and D’ peaks and the suppression of the 2D peak in the Raman spectrum suggest that a high concentration of defects was introduced during the deposition. According to XPS, the deposited vanadium was in the state V^5+^, which is the highest stable oxidation state of this element. As a result, an enhancement in the sensing properties of graphene towards the reducing NH_3_ gas was achieved. Such an improvement can arise from the strong adsorption ability of NH_3_ on V_2_O_5_ and possible redox reactions on the surface of V_2_O_5_, together with the activation of surface processes by using UV light illumination. The changing oxidation state of vanadium can modulate the electrical conductivity of a strongly coupled graphene–V_2_O_5_ system. Additionally, gas adsorption may be enhanced at the phase boundaries between a very thin nanostructured V_2_O_5_ layer and graphene substrate.

## Experimental

**Figure 6:**
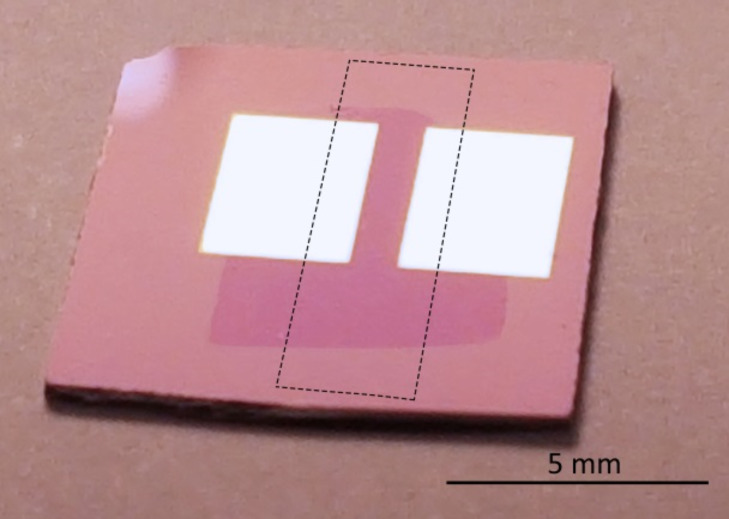
Photograph of a gas sensor device based on PLD-functionalised CVD graphene. The gap between the electrodes is 1 × 4 mm^2^. The graphene sheet on top of the electrodes can be seen due to its slightly different reflection properties. The area of the laser-deposited V_2_O_5_ is marked with the dashed box.

Graphene was grown on a commercial 25 µm thick polycrystalline copper foil (99.5%, Alfa Aesar) using a home-built CVD reactor. First, the foil was annealed at 1000 °C in Ar/H_2_ flow (both 99.999%, AGA Estonia) for 60 min, and then exposed to the mixture of 10% CH_4_ (99.999%, AGA Estonia) in Ar at the same temperature for 120 min. The sample was allowed to cool slowly, 15 °C/min in Ar flow. The as-grown graphene film was transferred onto a Si/SiO_2_ substrate by using poly(methyl methacrylate) (PMMA; MW ≈997,000 Da, GPC, Alfa Aesar) as a supporting material. The PMMA solution (1% in chlorobenzene) was spin-coated onto graphene/Cu, dried, and the Cu foil was dissolved in ammonium persulfate solution overnight. The floating PMMA/graphene film was rinsed with deionized water and transferred onto the Si/SiO_2_ substrate (see Figure 6) equipped with Pt electrodes (60 nm thick) that were deposited through a shadow mask by magnetron sputtering. The gap between the electrodes was 1 × 4 mm. The sample was dried in air and then heated on a hot plate to allow the PMMA film to soften, which improved the contact between graphene and the substrate. Then the PMMA layer was removed by dissolving in dichloromethane (Alfa Aesar).

In the PLD process, a ceramic V_2_O_5_ pellet was used as an ablation target. The sensor substrate was held in place by a shadow mask through which V_2_O_5_ was deposited onto graphene. A KrF excimer laser (COMPexPro 205, Coherent; wavelength 248 nm, pulse width 25 ns) was used for deposition. For the PLD target, a fine microcrystalline powder of V_2_O_5_ (99.6%, Aldrich) was pelleted at 740 MPa and subsequently baked at 550 °C in air for 5 h. Before starting the PLD procedure, the PLD chamber was evacuated and the sensor substrates were heated in situ at 150 °C for 1.5 h and then cooled down to room temperature. The heating procedure was established in order to clean the graphene surface and minimise the effect of trace contaminants left from the manufacturing process of the graphene sensor. The V_2_O_5_ target was ablated by using a laser pulse energy density of 5.0 J/cm^2^ in the presence of 5 × 10^−2^ mbar of O_2_. During the deposition, the sensor substrates were kept at room temperature, with other typical process parameters as follows: laser pulse repetition rate 5 Hz, number of laser pulses 120, and the distance between the substrate and the target 75 mm. Under exactly the same experimental conditions, another V_2_O_5_ layer was deposited onto a fused quartz substrate for the evaluation of the mass thickness of the deposited layer in the XRF experiment.

The structural characterization of graphene was performed by using a micro-Raman spectroscopic system (Renishaw, inVia) with an excitation wavelength of 514 nm. The vanadium concentration in the deposited layer was analysed with X-ray fluorescence (Rigaku, ZSX 400). The oxidation state of the deposited vanadium was also determined by X-ray fluorescence. The XPS spectra were acquired using a Scienta SES-100 spectrometer. The excitation source was a polychromatic twin-anode X-ray tube (Thermo, XR3E2) with Mg Kα (1253.6 eV) 300 W irradiation at an analyser–source angle of 45°. The semi-quantitative analysis was conducted using the methods described by Seah et al. [39] for quantitative XPS measurements. The spectra were energy calibrated to 284.8 eV (from 284.9 eV) using the C–C peak component of C 1s. The software used for peak fitting was Casa XPS (version 2.3.16).

The measurements of the electrical characteristics and gas sensitivity were carried out with a source meter (Keithley, 2400) in a sample chamber with a volume of 7 cm^3^ equipped with a gas mixing system based on mass flow controllers (Brooks, model SLA5820). The voltage applied between the electrodes was 100 mV. The gases used in our measurements N_2_, O_2_, CO/N_2_, NO_2_/N_2_, and NH_3_/N_2_ were all 99.999% pure. A synthetic N_2_ and O_2_ air mixture (79% and 21% in the mixture, respectively) was used as a carrier gas and the relative concentration of N_2_ and O_2_ gases was held constant during measurements. The gas flow through the sample chamber was kept constant at 200 sccm while the concentration of the test gas was regulated by the ratio of the flow rates of individual gas components. The nominal relative humidity of the testing gas was 20% during the measurements. All the sensor measurements were done at room temperature. The sensor system was exposed to light from a Xe/Hg high pressure lamp (Hamamatsu). The UV wavelength of 356 nm was selected with a narrow-band interference filter (Andover). The light intensity on the sample was 10–20 mW/cm^2^.
